# Social ecology of artisanal sand mining in the Niger River around Bamako, Mali

**DOI:** 10.1371/journal.pone.0318029

**Published:** 2025-01-30

**Authors:** Katharina Salomea Hemmler, Bouraima Camara, Andreas Buerkert

**Affiliations:** 1 Organic Plant Production and Agroecosystems Research in the Tropics and Subtropics, Universität Kassel, Kassel, Germany; 2 Faculté des Sciences et Techniques, Université des Sciences, des Techniques et des Technologies, Babalabougou, Bamako, Mali; Cranfield University, UNITED KINGDOM OF GREAT BRITAIN AND NORTHERN IRELAND

## Abstract

Sand, shaping both natural waterways and urban infrastructure, has recently seen a major surge in extraction, particularly in rapidly urbanizing regions like West Africa. To assess the organization, quantification, and socio-ecological implications of sand mining around Mali’s capital Bamako, we employed a mixed methods approach including structured and unstructured interviews, truck counts, turbidity analyses, and river depth measurements. Our study identified five artisanal systems for mining sand and gravel from the Niger River, using tied-up pirogues, single pirogues, carts, tractors, and trucks. Recent increases in extracted quantities, workforce size, and sand prices were observed, resulting in an estimated annual extraction of 4.86 million m^3^ in 2022, mainly sourced from upstream of Bamako. With extraction rates surpassing natural replenishment, the riverbed in the study communities of Gouni and Usine Toch has reportedly lowered by 1.4 m and 1.8 m during the last 50 years. Mining activities are highly informal, characterized by self-organization, low and irregular salaries, and unsafe working conditions, particularly for women. Economically, sand mining activities have created symbiotic relationships rather than conflicts with local farming, fishing and other livelihoods. Sand mining operations did not significantly affect the Niger River’s water turbidity, which varied primarily with seasonal rainfall fluctuations. Recent developments suggest that mining activities are accelerating, with mechanized practices likely to replace current artisanal methods and underlying social structures.

## 1 Introduction

Sand not only forms the basis of natural landscapes, shaping the intricate network of rivers and coastlines around the world, but also serves as the fundamental component of the built infrastructure in modern civilization. In the same way that we build houses made of sand to protect ourselves, the presence of sand in our environment is essential for the protection and resilience of nature [1]. As untreated sand from deserts is too fine and smooth for building purposes, construction sand and gravel are mined from rivers, coastlines, and terrestrial deposits for the construction industry to meet the infrastructural demand driven by urbanization and population growth. Although mined sand is primarily utilized locally, the rising demand for sand is a global phenomenon, particularly prevalent in Asia and Africa [2,3]. While in recent years industrialized countries witness a decline or reach a maintenance phase in sand extraction, the economically developing world is experiencing a rise or peak in sand demand [4]. In these regions, sand mining is often characterized as a small industry with high informality, making it challenging to monitor and regulate [2].

Sand mining can be categorized into active sources, such as rivers and deltas that naturally replenish through erosion and sedimentation, and passive sources, such as geological deposits [5]. Rivers are a significant source of sand and gravel due to their proximity to many cities resulting in low transportation costs, the natural process of rock grinding, and the production of angular-shaped minerals ideal for construction [6]. Hence, river sand mining is extensively practiced in Southeast Asia, India, and China, where unregulated extraction and the associated environmental effects have increasingly attracted media and research attention [7]. Across the globe, the identified environmental effects encompass morphological changes, such as channel incision, widening, and increased depth [8–11]. Additionally, alterations in sediment and flow characteristics, such as sediment or flow reduction, increased flow velocity, and changes in flow patterns, were reported, as were increased turbidity and saltwater intrusion [12–14]. Furthermore, effects on flora, such as the removal of vegetation and destruction of riparian habitat, and on fauna, including reduced fish reproduction, hindered fish migration, species extinction, and shifts from lotic to lentic species, were identified [15–18]. There were also effects on land, such as bank erosion or collapse, on air quality, including dust and noise pollution, and on surrounding areas in the form of lowered (ground)water tables [10,13,19]. The described environmental effects highlight the tendency for sand, considered a common resource with open access but limited availability, to be over extracted, resulting in a classical tragedy of the commons scenario in numerous countries [20,21].

In Mali, West Africa, urbanization is the main factor leading to increasing sand extraction, the total urban land area expanding by an average factor of 5.3 [22,23]. A notable example of this is Bamako, the nation’s capital and home to over three million inhabitants in 2024 [24]. It has experienced an increase in built-up area by 126% from 1990 to 2018 within the district and further stretched into surrounding suburbs [25]. Bamako’s expansion is driven by general population growth and rural-urban migration, which occurs seasonally during the dry season due to work-related migration, as well as permanently as a consequence of repetitive droughts and growing security issues. The growth of the urban population poses challenges related to basic services like safe water, sanitation, and electricity, as well as issues concerning traffic, drainage, and pollution [26].

To meet the infrastructure demands of Bamako’s spatial growth, substantial amounts of sand and gravel are required, sourced from the Niger River which is considered to be West Africa’s vital lifeline. Named the "great river" in the local Manding language, it fulfills crucial functions by providing and facilitating drinking water, irrigation, fishing, energy, and transportation [27]. At many locations along the Niger River bend and downstream, navigability is hindered by siltation, induced by wind or water erosion bringing sand from the Sahara Desert. This phenomenon, particularly prevalent during the dry season, poses challenges such as flooding and adversely affects fishermen and farmers reliant on adequate water levels. Consequently, sand mining activities are advocated in Malian media and public discourse as a measure to combat siltation [27,28]. However, the situation in Bamako contrasts with that of Central Mali, as the siltation from the Sahara Desert is minimal, and the ongoing mining operations have resulted in a deficit of sand, with extraction rates surpassing replenishment processes [29]. Despite the crucial role of sand and gravel in the physical expansion of Bamako, their extraction, as well as the associated societal and environmental effects, have not been properly addressed, neither scientifically nor politically. Scholarly attention to this issue has been scarce [29–31]. This aligns with a global pattern of inadequate scientific representation of sand mining activities, especially in the Global South, as well as artisanal mining, and the quantification of extracted sand [2,32].

Therefore, this study was conducted to address existing research gaps by (i) assessing the actors involved, their roles, processes, and the cost structures of diverse sand mining methods, (ii) quantifying the volumes and spatial dimensions of sand extraction, and (iii) determining the effects of sand mining on the environment, agriculture, fishing, and other livelihoods of relevant stakeholders.

## 2 Materials and methods

### 2.1 Study area

The Bamako District (12°38’33.2"N 7°59’56.2"W, 330 m above sea level) and the southern Koulikoro Region, which is encircling Bamako, are located in the tropical savanna a zone [33]. Their climate is characterized by a unimodal precipitation distribution with a rainy season from June to October. From 1991 to 2020, Bamako experienced an average annual precipitation of 947 mm (min: 751 mm—max: 1205 mm) and an average temperature of 28.3°C between 2018 and 2021 [34].

**Fig 1:**
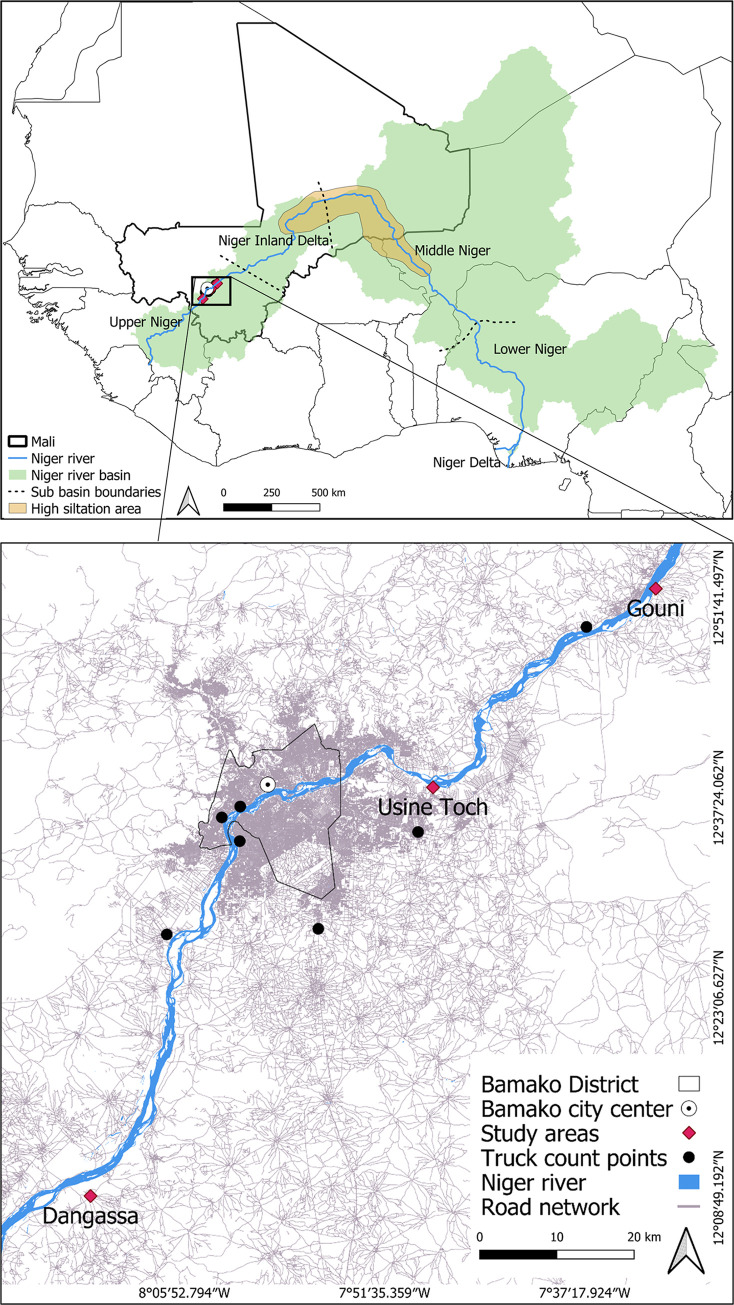
Location of the study areas in southwest Mali, West Africa [36,38–41].

The Niger River originates in the Fouta Djallon Plateau in the Guinea Highlands at an altitude of about 800 m. Spanning 4,200 km, it is the third longest river in Africa, after the Nile and the Congo. The river flows through four countries, including Niger and Nigeria, which are named after it, and its basin covers an area of 2.27 million km^2^, shared by ten countries. The river is typically divided into four sub-basins: the Upper Niger, the Niger Inland Delta, the Middle Niger, and the Lower Niger (Fig 1) [35]. As the river flows northeast into Mali, it reaches the Large Bend, where the gradient decreases and sediment is deposited into the Inland Niger Delta. This vast area of marshes, swamps, and pools is crucial for agriculture and fishing but is increasingly threatened by desertification from the encroaching Sahara [36,37]. Turning south and passing through Niamey, the capital of Niger, the river continues into Nigeria, where it makes two sharp turns known as the Nigerian Bends. Flowing through a subtropical environment that increases the water flux, the Niger joins with the Benue River before reaching the Niger Delta—one of the world’s largest deltas, spanning around 19 thousand km^2^. There it finally empties into the Atlantic Ocean at the Gulf of Guinea [37].

Our study focused on three communities along the Niger River within the Koulikoro Region, situated at beeline distances ranging 20 to 60 km from Bamako’s city center (Fig 1). Village selection was primarily based on the communities’ historical reliance on fishing and/or farming practices, complemented by the engagement in sand mining activities. This allowed an assessment of the interplay among these primary livelihood activities within the local population. The three chosen communities were:

1. Usine Toch (12°38’07.9"N 7°48’07.9"W), home to around 300 inhabitants, predominantly composed of the *Bozo* people, often recognized as the "Masters of the River" due to their fishing expertise.
2. Gouni (12°52’21.1"N 7°32’08.1"W), housing approximately 1,600 inhabitants, characterized by a mix of the *Bambara* ethnic group, predominantly farmers, and the *Bozo*.
3. Dangassa (12°08’54.4"N 8°12’36.0"W), a substantially larger community with 11,000 inhabitants, primarily consisting of the *Malinke* ethnic group known for their subsistence farming practices.

### 2.2 Data collection and analysis

A mixed methods approach, consisting of structured and unstructured interviews, turbidity analyses of the Niger river, river depth measurements and counts of sand trucks, was used to obtain insights into sand mining activities in the Bamako District and Koulikoro Region. In total, 85 individuals were interviewed, comprising sand miners, farmers, and fishers from Usine Toch (n = 28), Gouni (n = 29), and Dangassa (n = 28). The questionnaire encompassed three key sections: i) socio-demographic characteristics, ii) details on farming, fishing, and sand mining practices (acknowledging that not all were engaged in each of these), and iii) effects of sand mining on other livelihoods, the environment, and the community. The full questionnaire and responses can be found in S1 and S2 Files. Study participants were selected using a simple random sampling technique with the only selection criteria being their employment in farming, fishing, and/or sand mining. The gender balance was predominantly male, with 86% of respondents being men. This reflects the gender distribution across the surveyed sectors, with fishing being almost exclusively male, and both farming and sand mining predominantly male. The age distribution of participants ranged from 16 to 70 years, with an average age of 42 years. For data collection, the open-source software KoboCollect (version 2.021.47, KoboToolbox, 2022) was employed. The survey was translated from English or French to Bambara, potentially introducing a translation bias due to variations in language nuance and interpretation. Due to low literacy rates, informed consent was obtained verbally. All participants were read a standardized information script, and their verbal consent was documented electronically by the translator prior to the interview. Ethical approval was obtained from the central ethics committee of the University of Kassel prior to data collection which spanned from 11^th^ February to 17^th^ May 2022.

The unstructured interviews engaged a broad range of stakeholders from within the study communities and beyond. Participants comprised 11 sand mining association heads from the study communities (3) and other sand mining sites (8), 18 sand miners from locations outside the study communities (Katibougou, n = 7 and Kalabancoro, n = 11), one sand truck driver, and one representative each from key regulating agencies and political institutions. These included Koulikoro’s town hall, regional directorates of agriculture, water and forests, fishing, and the Belgian non-governmental organization (NGO) Enabel. Including different stakeholders allowed a more comprehensive understanding of sand mining activities by gathering diverse perspectives as well as insights from workers across the different mining locations and systems.

To estimate the volumes of mined sand, we recorded truck counts at seven specific points strategically positioned to encompass major sand flows into the city (Fig 1). Truck count points were placed along all main truck routes to ensure comprehensive coverage without double-counting, including sites near newly developing suburbs like Koulikoro and close to primary unloading areas such as Kalabancoro. This placement was critical as trucks were nearly guaranteed to use these main routes, given the poor condition, lack of paving, or narrow width on most smaller roads. Specifically, the points included Banankoro (12°28’3.073"N 7°56’19.163"W), Farabana (12°27’38.766"N 8°7’8.483"W), Tiéguena (12°34’57.847"N 7°49’11.659"W), Kalabancoro (12°34’18.892"N 8°1’55.707"W), Sebenikoro (12°36’0.832"N 8°3’12.612"W), Djikoroni (12°36’47.265"N 8°1’53.595"W), and Koulikoro (12°49’36.685"N 7°37’10.120"W), with truck sizes from 3.5 m^3^ to 33 m^3^. Data collection spanned 15 days each (early morning to late evening, as no trucks were allowed to enter the city at night), with measurements conducted in May, July, and October to cover the main mining period and account for potential seasonal variances in sand transportation quantities. Truck count data and calculations can be found in S3 File.

Water samples were collected to evaluate the influence of sand mining activities on water turbidity as a water quality and aquatic habitat indicator within the Niger River using a PCE-TUM 20 Turbidity Meter (PCE Instruments, PCE Deutschland GmbH, Meschede). Turbidity measurements were conducted at various points (100 upstream, 100 m downstream and 300 m downstream of sand mining activities; at the left, center and right side of the river, and at two depths: the water surface and one meter depth) within the communities of Usine Toch and Gouni. Dangassa was omitted from this analysis due to ongoing riverine gold mining activities in the area that could have significantly influenced the study’s results. The temporal scope encompassed a year-long period, from March 2022 to February 2023, with measurements undertaken every two months. Three subsamples were taken at each location to account for measurement errors and to enhance accuracy of the data collected. In total, the 648 subsamples were condensed into 216 measurements by averaging the three subsamples at each sampling point and depth. Measurement data can be found in S4 File. To test for significance in water turbidity levels, a Liner Mixed Model (LMM) was fitted. Tukey’s Ladder of Powers was used to transform skewed data distributions. The nested ANOVA designated mining status and month (and their interaction) as fixed effects and community, river position (left, center, right) and depth (surface, 1 m) as random effects to account for potential variability between communities, river positions, and depths, thereby improving the robustness of the analysis [42,43].

Additionally, a river depth analysis was carried out in Usine Toch and Gouni. To accomplish this, we crossed the Niger River by pirogue and, at regular intervals ranging from every 20 to 50 meters, used a long rope marked at each meter with a heavy metal piece attached to measure the depth. Retrospective data was gathered from community members who remembered being able to walk across the river during the dry seasons of 1974 in Gouni and 1976 in Usine Toch. From this data (see S5 File), we calculated the riverbed area using the program 3DExperience Catia (version CATIA R2024x, Dassault Systèmes, France, 2024), which facilitated modeling the points into a surface to accurately calculate the area. By comparing the calculated areas from 1976/1974 to those of 2022, we quantified the volume of sand extracted that exceeded natural replenishment rates, resulting in a lowering of the riverbed. By dividing the total area by the number of years, we determined the average annual riverbed lowering for the two study communities. Descriptive statistics, t-tests, ANOVA, and Pearson’s Chi-square test were performed using the R 4.1.2 software, RStudio, and the Tidyverse package [43].

## 3 Results

### 3.1 Sand mining systems

**Fig 2:**
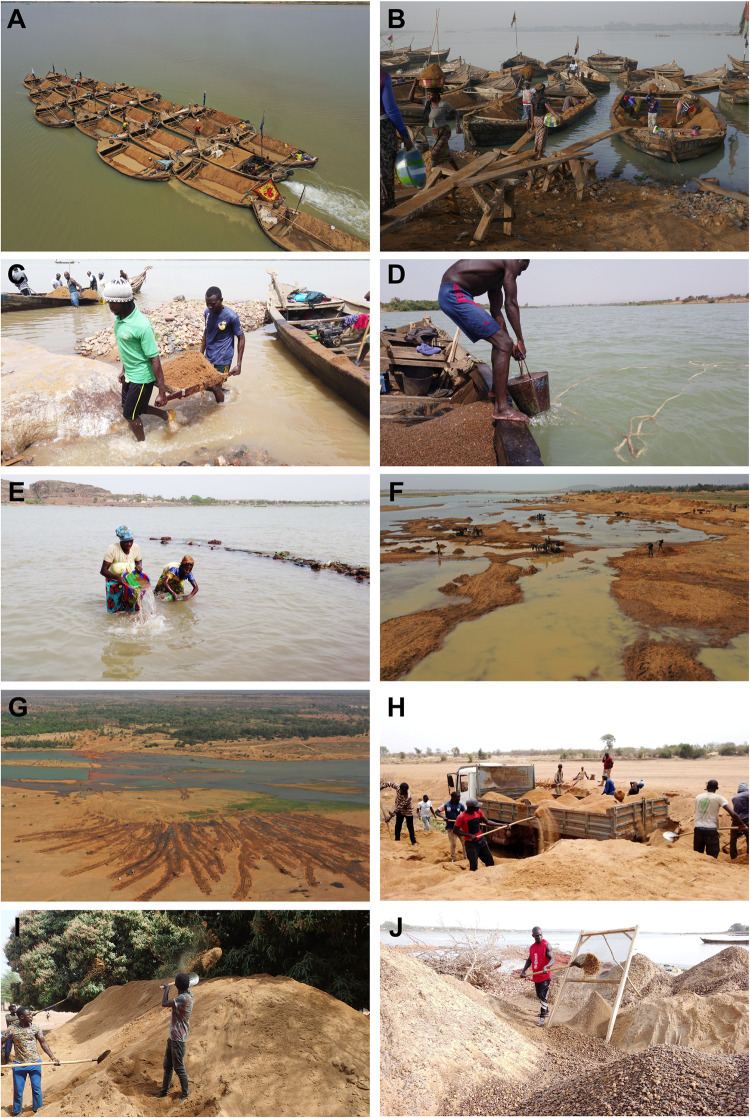
Sand mining types practiced around Bamako, southwest Mali. A. Tied-up pirogue system, B. Unloading with buckets, C. Unloading with trays, D. Single pirogue system, E. Collection of fallen sand, F. Loading carts pulled by donkeys, G. Extraction by tractor, H. Direct loading of truck, I. Stocking sand, J. Sieving gravel.

Sand mining along the Niger River near Bamako encompasses the extraction of three types of sand, differentiated by size and usage (small grains for tiling and finishing in masonry work, medium for brick laying, and large for all-purpose construction, hence the most popular), as well as gravel (for setting building foundations and roads). The process involves various manual and artisanal procedures of in-channel mining, defining five sand extraction systems:

The first system relies on tied-up pirogues (Fig 2A), a group of wooden boats tethered together with one engine positioned at the rear, propelling them collectively. Such fleets were observed to operate upstream of Bamako, with the starting/unloading point ranging from the city center to Banko Koura (25 km south of Bamako). Typically, the boat owner was responsible for paying the engaged workforce, including the engine operator, sand divers (one person per pirogue, using buckets), sand unloaders (either a group of 4–8 women using buckets, Fig 2B, or 3–6 men using trays, Fig 2C), and truck loaders (using shovels), and in return received the revenues generated through the subsequent sale of sand to truck drivers. During the dry season, the levels of the Niger River were low, posing a risk of collision with rocks. To mitigate this risk, the boats were only loaded to half their capacity, with up to 20 pirogues tied together for the 24-hour round trip in 1.5 m deep water from Bamako to the area of Djioliba and back. During the later part of the rainy season, higher water levels created more favorable waterways. Boats were then heavily loaded, with up to 30 pirogues tethered together. There, the current was stronger, the depth for diving increased (to 2 m), and the sand extraction area shifted further upstream, resulting in an extended trip duration of 48 hours for a round trip from Bamako to the area of Dangassa and back. The tied-up pirogue system was the most practiced during the rainy season, making it the main extraction period of sand around Bamako.

The second type of sand mining was based on single pirogues operating individually (Fig 2D), each equipped with its own engine allowing high mobility. These pirogues were utilized both upstream and downstream of Bamako, and common in the study areas of Usine Toch and Gouni. Working in teams of two, one person assumed the role of the diver, while the other pulled up the loaded buckets. Mining depths could reach up to 15 (20) meters in the dry (rainy) season around Usine Toch and the distance to the unloading point was short, allowing for several trips per day. This system was practiced all year round, whereby the trip duration increased during the rainy season, e.g., in Gouni from less than two hours per trip to around three hours. In contrast to the tied-up pirogue system, the single pirogue system was less active during the rainy season due to the increased diving depth and time per trip, the poorer road infrastructure, and competing farming activities. Both systems were complemented by the collection of sand at unloading points (Fig 2E). Collection of accumulated sand was primarily undertaken by women, utilizing (perforated) buckets for sand extraction and piled collection.

The third system was practiced in Katibougou (near Gouni/Koulikoro, 60 km beeline to Bamako) and Dangassa on the shore of the Niger River. It required low water levels and was therefore only practiced between February and July. Here, the sand was directly extracted from the semi-dry parts of the riverbed. Shovels were used to loosen, pile up, and load sand onto carts pulled by donkeys (Fig 2F). Sand collection points were close to the shore, just far enough to be above the water line and accessible by trucks.

In Katibougou during the dry season, sand mining operations involved the use of tractors with trailers and motorized tricycles (Fig 2G). These vehicles accessed the semi-dried riverbed directly, utilizing shovels to load the trailers. The extracted sand was then either stockpiled for subsequent pick-up by trucks or directly delivered to local customers.

The fifth sand mining system was observed in Dangassa and involved the direct loading of sand onto trucks of 4–15 m^3^ (Fig 2H) from the semi-dried riverbed. A pathway was created by laying out branches to facilitate truck access. Truck loading was performed by a group of up to 8 men using shovels, within a span of 10 minutes. The extracted sand was either collected in piles for pick-up by larger trucks or delivered directly to customers. Notably, this system operated exclusively during the dry season when the riverbed was devoid of water. During the rainy season, the riverbed was filled with water, and sand mining activities were conducted by tied-up pirogues.

In all systems (with the exception of the direct sale of sand of the tractor- and truck-system), the sand was manually unloaded of the pirogue/cart/tractor/truck, piled up, and loaded onto trucks for delivery. This process allowed for stockpiling and selling based on demand but contributed to an overall low labor-efficiency of the sand extraction process. During the dry season sand was stockpiled in higher quantities for the rainy season (Fig 2I). Causes for this were (a) the demand for sand increasing during the rainy season as construction was facilitated by the availability of water (particularly towards the end of the rainy season when the risk of torrential rainfall was reduced) and (b) sand was accumulated particularly in the Koulikoro area due to seasonal fluctuations in sand mining activities. High mining quantities during the dry season were met with restrictions during the rainy season due to increased water levels, necessitating sand storage to guarantee sand supply throughout the year.

There were two mining systems for gravel: first, as it is deposited below the sand layer in the riverbed, gravel was mined in the same way as the single pirogue system described above. It was either utilized directly or, if extracted as a sand-gravel-mixture, sieved through a standing mesh (Fig 2J). Due to the increased diving depth and sedimentation of sand during the rainy season, gravel was primarily extracted during the dry season. The second method was practiced around the Dangassa area and involved gold mining dredges. Hereby, gravel was obtained as a by-product of gold dredging, loaded directly from the dredge belt onto the pirogue.

### 3.2 Scope and development

Throughout the year, the various sand mining systems contributed at different intensities to fulfill the demands for construction materials of the rapidly expanding capital Bamako. Truck counts revealed that between May and October 2022, 64% of the sand originated from upstream sources, whereas 36% were mined downstream of Bamako. An estimated 4.86 million m^3^ of mined sand was calculated for 2022, based on cumulative data from May, July, and October, while factoring in seasonal variations of the extrapolation of the remaining months assuming peak extraction from June to October, a subsequent decline from November to January and an increase from February to May. Based on an assumed sand density of 1.88 g/cm^3^ of wet sand, the estimated quantity of sand transported annually to Bamako amounted to 9.14 million t.

Over the past few decades, sand mining increased throughout the various sites around Bamako-Koulikoro. According to sand mining association heads, 30 to 55 years ago, mining activities commenced at a modest scale, and have witnessed a gradual increase, particularly accelerating in recent years. In 1991, fewer than 20 pirogues operated in Kalabancoro, central Bamako, according to the association head, one of the pioneers of sand mining in the area at the time. However, this number surged to approximately 1,000 at the same extraction site 30 years later. As a result of persistent increasing mining, the sand extraction area has shifted over the years. In the early 2000s, sand extraction from the Niger River near Sebenikoro (less than 10 km from the city center) was feasible. Since then, the sand frontier has progressively advanced, currently spanning 40–60 km upstream of Bamako. Consequently, the dynamics of sand mining have resulted in three distinct but intertwined developments: the gradual shift of the sand extraction area over the years, the alternating shift of the mining area between the rainy and dry seasons, and an increase in river depth.

Within our study region, the heads of sand mining associations reported a steady rise in sand mining quantities over the decades, with a notable recent acceleration in Gouni and Dangassa. Most of the respondents noted increases in both the number of people engaged in sand mining activities and the quantities of sand mined, as well as a rise in sand prices, compared to 5–10 years ago, along with extraction rates exceeding the natural replenishment rates in the study communities (Table 1).

**Table 1:** Overview of workforce, sand extraction, and price trends in the study communities around Bamako, Mali.

	Workforce increase	Increase of extracted sand quantities	Extraction rate surpassing natural replenishment rate	Sand price increase
Affirmation	63%	61%	64%	73%
Negation	22%	30%	28%	13%
Uncertainty	11%	9%	8%	15%

However, variations were observed within the study communities: affirmation for the four aspects was lowest in Dangassa at 50%, 38%, 50%, and 29% respectively, compared to Usine Toch (59%, 61%, 56%, 90%) and Gouni (86%, 73%, and 80%, 69%).

**Fig 3:**
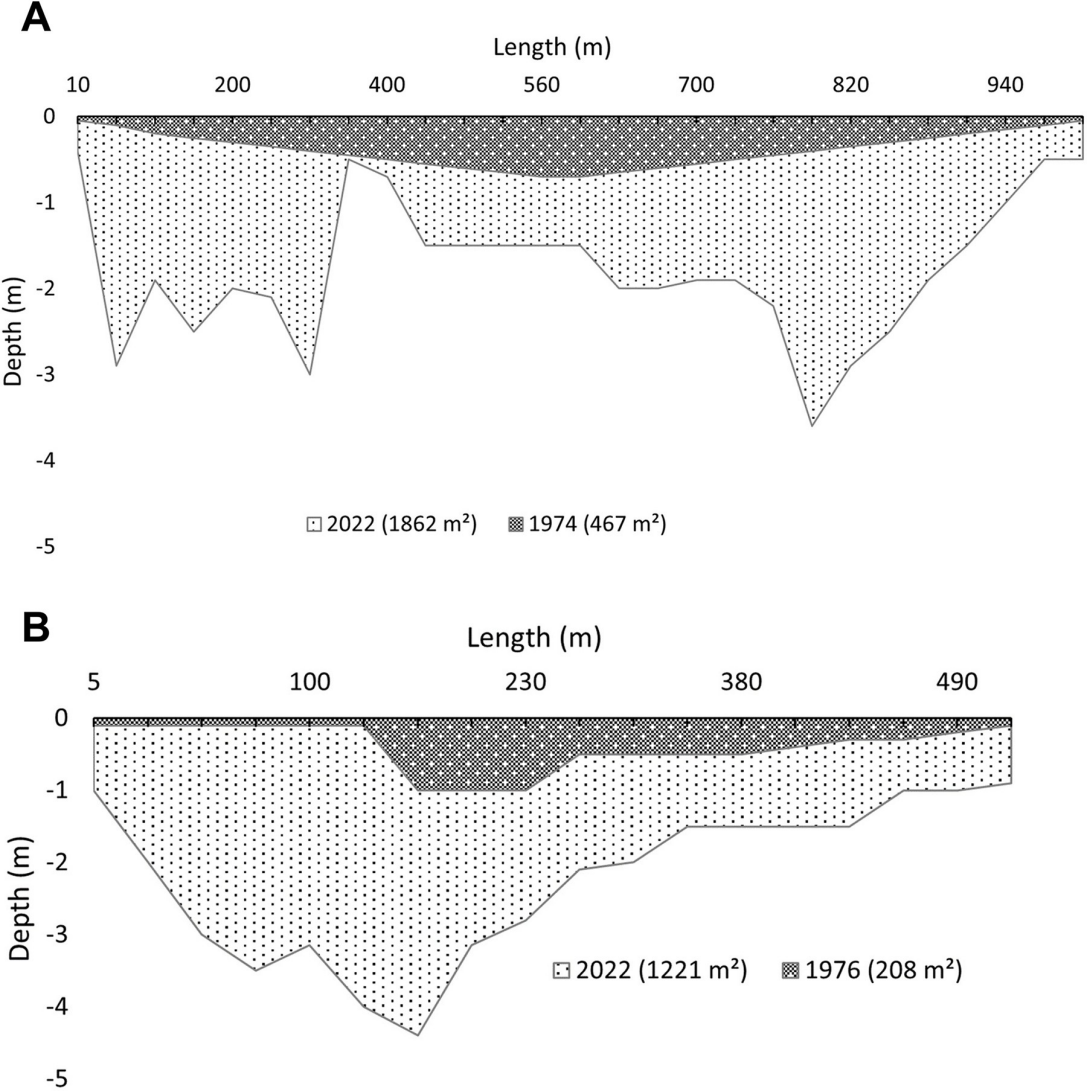
Putative cross-section of the Niger River in 1974/1976 and 2022 in two study communities. A: Gouni, B: Usine Toch, Koulikoro Region, southwest Mali.

A comparative analysis of river depth and historical data in Gouni and Usine Toch revealed a clear difference in the depth of the riverbed and cross-sectional area of the Niger River between the periods 1974/1976 and 2022. There was a total average riverbed lowering of 1.4 meters in Gouni and 1.8 meters in Usine Toch, representing a yearly average cross-sectional reduction of 2.9 cm (Gouni) and 4.0 cm (Usine Toch) or a total cross-sectional reduction of 1395 m^2^ (Gouni) and 1013 m^2^ (Usine Toch, Fig 3).

Concerning the future trajectory of sand mining, the majority of interviewees (83%) anticipated a continuation of mining activities, while 11% expressed uncertainty, and 6% rejected the prospect of ongoing mining operations. However, none (0%) of the respondents expressed a desire for their children to pursue a career as sand miners, with 15% preferring farming, 3% fishing, and 82% indicating a preference for alternative professions.

**Fig 4:**
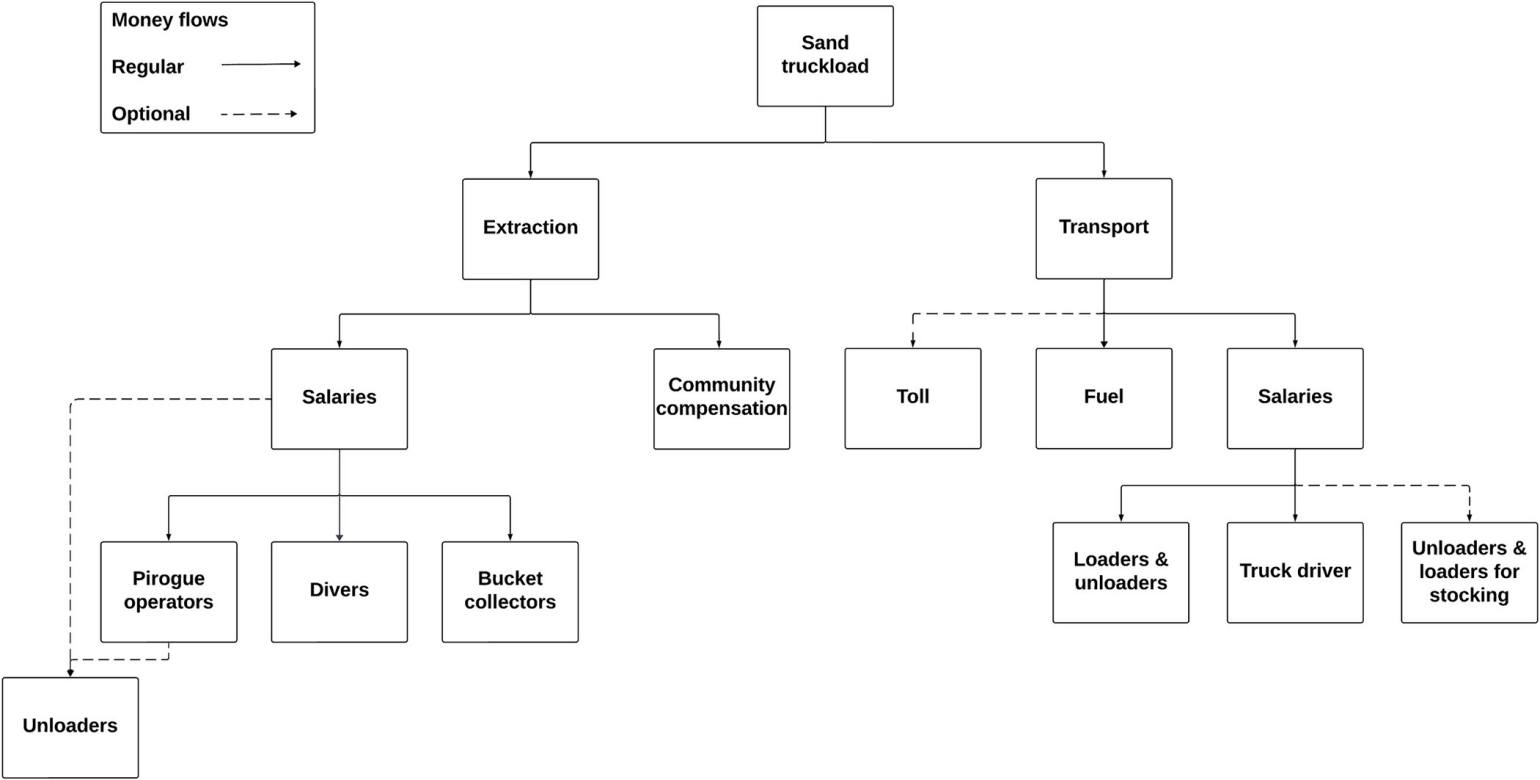
Money flow structure of pirogue sand mining around Bamako, Mali.

Payment systems, actor salaries, and pricing dynamics are intricately tied to and influenced by the fluctuating demands in the sand market. As of 2022, diverse payment systems were in place across the different sand extraction sites, involving several cost factors on both the extraction and the transportation side (Fig 4). Salaries were paid by the boat owner / manager to pirogue operators, divers, and women collecting sand with buckets (based on the size of the sand pile). Unloaders were either compensated directly or by pirogue operators (e.g. in Usine Toch two operators received 7,500 FCFA (= 11.43€ [44]) to 10,000 FCFA per trip, from which 1,200 to 1,500 FCFA were handed to unloaders). The community compensation took various forms: In Gouni, each truckload incurred a fee of 1,500 FCFA, with 500 FCFA allocated to the village head, 500 FCFA to the town hall, and the remaining 500 FCFA to the association. In Usine Toch, a monthly collection fee from boat owners was levied for joint payment to the town hall. It varied from 2,000 to 10,000 FCFA per working group to which 2,000 to 2,500 CFA were to be added for the village head. In Dangassa, the payment depended on the destined location: for trucks transporting sand to Bamako, the initial truckload of the day had to be delivered to the community. If the sand was sold or deposited in Dangassa, a payment of 5,000 FCFA was to be made to the community. For pirogues, the fee was 1,000 FCFA per pirogue in 2022 and doubled by 2023. Cart operators paid 10,000 FCFA to the village annually. The subsequent sale of sand to truck drivers varied by locality and grain size: a 15m^3^ truck load was sold in Usine Toch at 75,000 to 80,000 FCFA during the dry season, whereas in Gouni, the range was 25,000 to 30,000 FCFA. For transportation, payments were required for fuel and salaries for the truck driver, loaders, and unloaders. Additionally, if the sand was stockpiled and sold during the rainy season, the price would increase due to the need for extra loading and unloading. Moreover, road toll fees were charged on certain routes; for example, a fee of 1,000 FCFA was levied upon exiting the town of Koulikoro. Considering these expenses, a truckload of sand was priced at approximately 60,000 FCFA for 7 m^3^ or 110,000 FCFA for 15 m^3^ in Bamako.

### 3.3 Working in sand mining

In the study communities, 54% of the respondents were involved in sand mining activities, undertaking diverse roles such as supervision, sand diving, pirogue operation, sand unloading, cart loading, truck loading, sieving, and collecting fallen sand with buckets. 59% of the sand miners did not engage in any other occupation prior to their employment in mining. The primary motivations for an involvement in sand mining were monetary gains, cited by 32 respondents, to support their families, invest in agriculture during the rainy season, and achieve financial independence. Additionally, 11 respondents highlighted the lack of alternative employment opportunities, particularly during the dry season, as a significant motivator. Family members engaged in sand mining were mentioned by 5 individuals, while 3 respondents cited other reasons. Income opportunities were for 87% of the sand miners the predominant positive aspect of sand mining.

The sand mining sector was highly informal whereby sand miners were typically self-organized at individual mining sites, and the formation of sand mining associations was common. These associations were generally organized locally per site, although there were exceptions, such as the presence of two associations in Kalaban Koro and a joint association of Kalaban Koro, Farabana, and Banko Koura. Each of the three study communities had a sand mining association, led by an association chief who was selected by the miners or appointed by the village chief. However, despite the presence of these associations, only 26% of the respondents reported being a member of such an organization. The lack of social protection measures, low salaries and irregular incomes were highlighted by approximately half of the respondents who reported erratic profitability due to price fluctuations and high market volatility. Safety and health concerns were also raised, whereby 22% of sand miners were concerned by the physical hardship of the work. Incidents of drowning sand divers were reported, and inadequate sanitation facilities further contributed to unsafe working conditions. Extended working hours were prevalent at an average of 5.9 weekly working days. Two thirds of the sand miners adhered to daytime working hours from 3–18 hours (averaging 8 hours per day). One third had flexible working hours, including day and night work, such as a 24–48 hour trips with the tied-up pirogue system or on-call duty for on demand sand delivery. The sector’s informality was further reflected in low levels of labor skills, characterized by a low educational background (only half of the sand miners had visited a French speaking school, 20% a Coran school and 30% no school at all). Commencing sand mining operations required only minimal investments, such as for a shovel or a bucket, and no formal permits were necessary. Informality was also acknowledged by authoritative bodies, such as the regional directorate of waters and forests and the town hall of Koulikoro, who described the sector as unprofessionalized and noted widespread tax evasion. Despite its undocumented nature and lack of governmental permission, sand mining was not perceived as illegal. About 90% of respondents asserted that there was no illegal sand mining, while the remaining 10% indicated that extraction was prohibited if prior notice was not given to other sand miners and association heads.

The NGO Enabel identified several gender-specific challenges that disproportionately affected female workers within the sand mining industry. Women were often marginalized and excluded from decision-making processes, such as setting prices, and encountered difficulties in securing a place to store sand. Furthermore, women were more susceptible to skin problems due to their involvement in collecting leftover sand and spending extended periods in the water. Women were also reported to be victims of verbal and sexual violence within the sand mining community. Additionally, the existence of shared toilet facilities, with men holding the keys, potentially made women reluctant to request access. Moreover, the town hall of Koulikoro and sand mining association heads highlighted the reduced profitability for women in the industry, particularly for those engaged in fallen sand collection using buckets.

### 3.4 Environmental effects of sand mining

**Fig 5:**
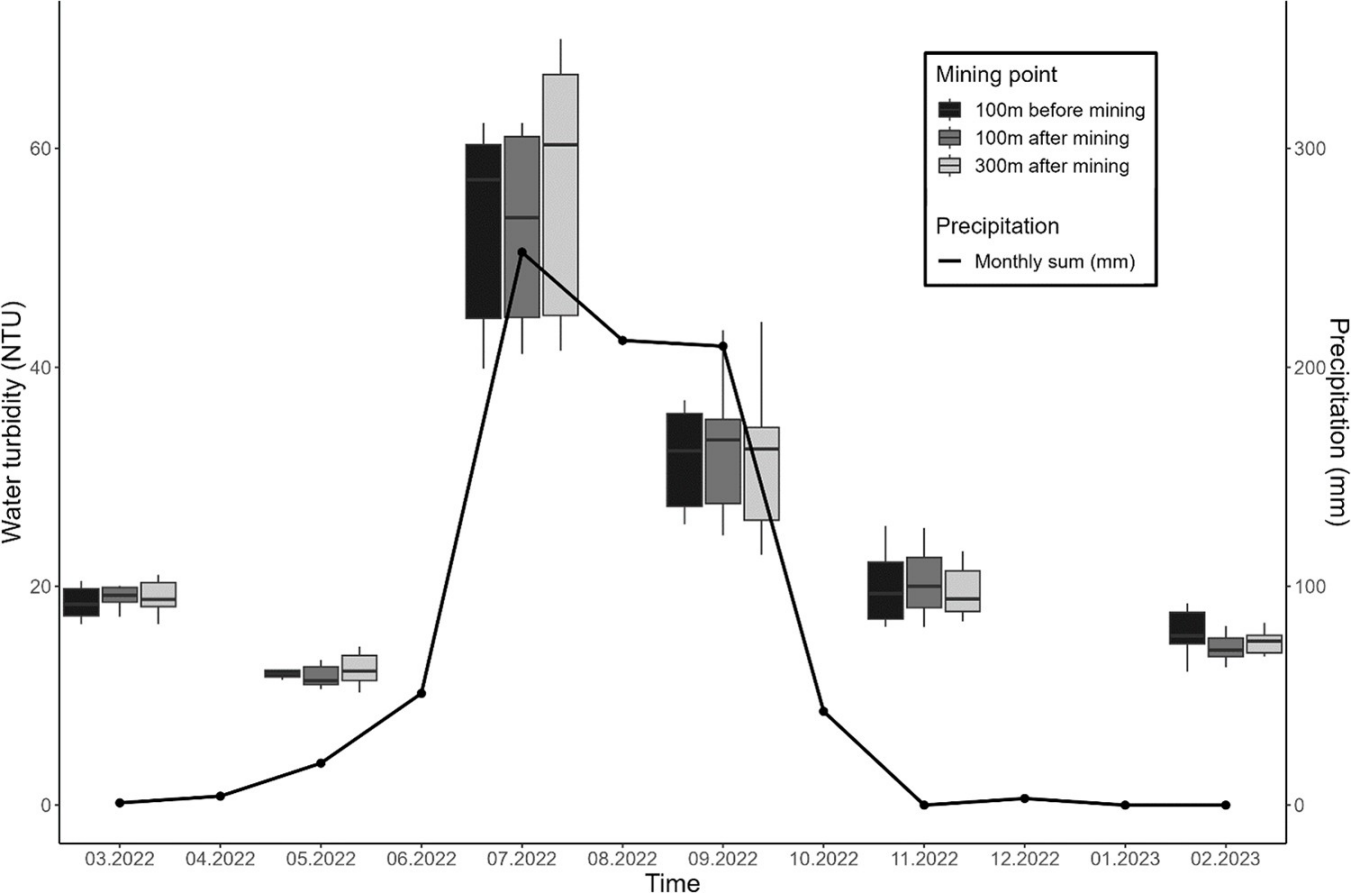
Bimonthly distribution of water turbidity in the Niger River in the study communities of Usine Toch and Gouni and monthly precipitation in Koulikoro, Katibougou, from March 2022 to February 2023, southwest Mali. Boxplots show the median, the 25% and 75% quantiles with whiskers marking up to 1.5 of the inter-quartile range. Source: Mali Météo, 2023.

The turbidity measurements conducted in the Niger River within the study communities of Usine Toch and Gouni yielded 10 to 92 Nephelometric Turbidity Units (NTU, average 26). The results from the LMM analysis indicated that sand mining did not have a statistically significant effect on water turbidity (p = 0.934; Fig 5). The interaction between month and mining status did not show a significant effect on water turbidity (p = 0.918). Turbidity averaged 26.5 (s.e. ± 2.1) at 100m before mining locations, 25.4 (±1.7) at 100m after mining locations, and 25.9 (±1.9) at 300m after mining locations. Season emerged as the single decisive factor for substantial fluctuations in river turbidity (p < .0001). The observed pattern revealed a rise in turbidity levels during the rainy season, followed by a decline during the dry season. Average turbidity strongly varied across seasons, with values ranging from 13.2 (±0.6) in May to 56.5 (±2.1) in July (5).

Only a small percentage of respondents (4%) reported mining effects on water quality, while 14% observed changes in water levels and 9% noted alterations in flow velocity as a consequence of sand mining. The majority of the interviewees (62%) indicated that sand mining did not adversely affect any of the riverine parameters. The respondents ascertained a change in water quality (n = 16), water levels (n = 12), and flow velocity (n = 14) attributed to gold mining activities rather than sand extraction. Similarly, only 4% affirmed an increased level of erosion into the Niger River due to sand mining, with 82% negating this effect, and 14% providing no response. Most respondents (75%) reported no negative effects of sand mining on terrestrial flora and fauna, while 14% did not provide an answer. About 11% mentioned as negative consequences of sand mining the cutting down of trees and branches to create roads on the sand in Dangassa during the dry season, as well as the habitat destruction of wild animals on river islands. The regional Directorate of Waters and Forests also claimed disturbance of aquatic animals, such as the hippopotamus (*Hippopotamus amphibius*). Additionally, the Koulikoro town hall noted a degradation of the ecosystem with adverse effects on aquatic plants and the destruction of riverbanks attributed to sand mining.

### 3.5 Interplay of sand mining, agriculture, and fishing

The study communities were defined by an activity mix of sand mining, farming, and fishing. While agriculture and fishing played an important role for many years (on average the respondents were farming for 29 years and fishing for 35 years), sand mining was a rather new livelihood strategy (practiced for an average of 11 years).

Over three quarters of the interviewees were engaged in agriculture, cultivating an average field size of six hectares, primarily with staples such maize, sorghum, millet, and rice. The main growing period was linked to the rainy season and stretched from June to October. During the dry season, only small-scale agriculture (gardening) for vegetable production along the Niger river and canals was practiced. The majority of the fields were characterized by rainfed cultivation (87%) and subsistence farming: 79% of the fields were cultivated exclusively for household consumption, while 15% met household needs and generated income through sales. The remaining 6% were sales oriented, catering to local markets and those in Bamako. Also, 86% of the farmers indicated that their field sizes have expanded in recent years, driven by factors such as growing family numbers (n = 26), declining soil quality (n = 12), economic considerations (n = 11), and other reasons (n = 7). Farmers reported secure access rights of their fields as the majority (76%) was owned by the farmer him/herself, followed by 10% belonging to family members, and 13% to a third party. The lack of mineral fertilizer, machinery and rainfall were named as the greatest difficulties of farming in the studied communities.

Around Bamako, the intertwining nature of agriculture and mining manifested itself several-fold. In terms of employment and livelihood strategies, farmers integrated sand mining with their crop production, with almost half (46%) of the farmers engaging in sand mining. Most of the interviewees (73%) reported no effect of sand mining on agricultural activities, as the mining was mainly carried out during the dry season as opposed to the main agricultural production during the rainy season. Thus, the respondents engaged in both activities with no reduction in the availability of the agricultural workforce (82%). However, it should be noted that in other communities, particularly in the city center, the situation differed. Many young people who had migrated from Northern Mali settled in Bamako permanently and did not return during the rainy season, resulting in a shift of the workforce from agriculture to participation in sand mining. In terms of financial dynamics, a positive synergy between the two sectors was identified. Respondents highlighted the beneficial effect of sand mining on the size of fields (44%), crop yield (56%), and the quality of agricultural produce (42%). This was attributed to the supplementary monetary resources from sand mining that were invested in agricultural inputs. The representative from the regional directorate of agriculture also acknowledged this symbiotic relationship, emphasizing how the additional income from sand mining contributed to the enhanced purchasing power and nutrition of farming households. The mentioned adverse effects were the increased erosion of riverbanks where gardening was practiced, and the competition for land along the river.

15% of the respondents within the study communities participated in fishing, displaying increased engagement during the rainy season. Fishing was conducted for both household consumption and sale, targeting local markets and Bamako. The encountered challenges encompassed a decrease in fish stocks across all species attributed to overfishing and habitat destruction, with specific mention of the effect of the Sélingué dam construction, plastic pollution, and the presence of gold mining dredges. Additionally, fishers faced low prices for their catch due to the absence of adequate cooling storage facilities and reported difficulties in accessing essential materials such as bait.

The interconnection between sand mining and fishing was prevalent, with 38% of the fishers in the studied communities also engaging in sand mining. Parallel to the effects on agriculture, the influence on the accessible workforce for fishing varied. In the study communities, sand mining and fishing were executed during different times of the day or seasons (sand mining during the dry season and fishing at dawn and during the rainy season). In other areas, the seasonal return of migrants who had settled in Bamako to engage in sand mining activities may be uncertain. The majority of interviewees (86%) perceived no discernible effect of sand mining on fishing, while the remaining 14% identified specific effects, including the investment of sand mining proceeds in fishing materials, the adverse influence of sand mining activities in Dangassa, where the placement of sticks on the sand for truck access impaired throwing out nets for fishing during the rainy season, and the increased water depth on the river shores, rendering seine fishing unfeasible. The representative from the regional directorate of fishing attributed the decline in fish stocks partially to sand mining activities, citing mining-related factors such as the degradation of riverbanks and the destruction of aquatic fauna. Predominantly, non-mining-related contributors to the diminishing fish stocks were identified, including unsustainable fishing practices such as the use of close-meshed nets, improper cultivation practices such as gardening and farming in close proximity to riverbanks. The latter was perceived to cause erosion that washed fertilizers and other substances into the water, as well as the detrimental impact of gold mining activities, particularly the use of mercury. Furthermore, the creation of holes serving as spawning grounds for fish was identified as a positive effect of sand mining on fishing activities.

### 3.6 Sand mining and the community

Interviewees reported negative effects of sand mining on infrastructure, with 52% affirming a detrimental impact on road conditions, while 29% reported no negative effects and 19% did not respond. Noise pollution from sand mining operations was a problem for 55% of the respondents, particularly night-time honking. Communities responded to these challenges by implementing various solutions. In Dangassa, a new road outside the village was created after an incident with a truck, diverting the traffic away from the community to minimize noise pollution and strengthen residents’ safety. Similarly, in Usine Toch, road closures were implemented during the rainy season to mitigate the effects of mining related traffic on the roads, while in Gouni, funds were collected for road repair initiatives. The town hall of Koulikoro and truck drivers also acknowledged the deterioration of road infrastructure due to sand mining activities, further mentioning sand congestion of the road and water drains. On a positive note, sand miners contributed to the repair of roads, and occasionally trucks provided transportation for villagers to Bamako or nearby towns, such as children commuting to school. Additionally, the availability of sand from mining operations facilitated the construction of houses within the communities.

Negative effects on health were reported by a minority of respondents, whereby 29% noted health issues solely among workers and 11% acknowledged health concerns affecting community members in general. Health issues related to dust exposure were affirmed by 58% of the respondents. According to the regional directorate of fishing, the increased river depth and the presence of holes in the riverbed posed risks to individuals within the communities, predominantly children. Conversely, the positive outcomes resulting from sand mining were noteworthy, with the influx of income contributing to improved nutrition of community members. The reduction of poverty was also recognized by the regional directorate of waters and forests. Moreover, the regional directorate of fishing emphasized the role of sand mining in addressing unemployment and creating a melting pot by fostering social cohesion among diverse ethnic groups. Similar to farming and fishing, other livelihoods benefited from the financial resources generated by sand mining, with investments directed towards small-scale trading, animal herding, and butchery for instance. Additional sources of income were observed at the sand pits, including the sale of food, beverages, and herbal medicine, further diversifying the economic activities within the community. Additionally, the engagement in sand mining was found to deter individuals from participating in unsustainable practices such as deforestation, exemplified by cases where people opted to join sand mining instead of cutting down trees, as noted by the regional directorate of waters and forests.

## 4 Discussion

Manual sand mining is frequently linked with artisanal practices, yielding relatively low quantities of sand [45,46]. However, our results show that the sand miners around Bamako extracted considerable amounts of sand. Our annual estimate of 4.86 million m^3^ significantly exceeds previous estimates by Ferry et al. [29] which suggested extraction rates from 15 to 20 million m^3^ from 2000 to 2006 (equivalent to 2.5 to 3.3 million m^3^ per year). Similarly, Filho et al. [15] estimated that annually 5 to 10 million tons of sand were extracted in the country of Mali, in contrast to our calculation of 9.14 million tons for only Bamako and its surroundings. It needs to be noted that our estimation is likely conservative as some trucks may have day-transported sand via smaller roads or in close proximity to the sand source, such as used locally in Koulikoro, and were therefore not included in our calculations. Some minor sand smuggling may have also occurred at night when no counts were made or via small side roads even so both are unlikely to occur in any significant number given that the heavily loaded sand trucks must use well surveyed, solid (paved) roads when entering the densely populated city area. Overall, we are confident that less than 10% of the total sand flow remained unaccounted for. On the other side, the official records of trucks passing the city of Koulikoro, as shared by the Koulikoro town hall, only amounted to 27% of our own local counts. This reflects a substantial loss of tax income for Koulikoro. When combining our calculation with expected urban growth rates of Bamako [47], we may assume that the amount of extracted sand will increase during the next years and will from 2025 onwards easily surpass the 10 million t mark per year.

At the national level, Mali’s cement production in 2019 reached 660,000 t [48]. Considering the commonly practiced cement to aggregate ratio of 1:14 in the construction sector around Bamako (as opposed to the common ratio of 1:5 to 1:7), this would at the country level require approximately 9.31 million tons of sand. Remarkably, this aligns closely with our earlier calculation of 9.14 million tons for Bamako alone. However, the national demand for cement was estimated to be 3 million t [49], implying a sand and gravel requirement of 42.3 million tons nationwide—nearly 4.6 times our calculated estimate for Bamako.

At present, Bamako’s sand extraction rates are comparable to those of Ghana’s capital Accra, amounting to 8.4 million t per year [50]. However, other countries and regions exhibit higher annual demands and extraction rates for sand, such as an estimated 40 million m^3^ for Lagos, Nigeria [51], around 20 million m^3^ for Morocco [52], and 15 million t for the Brazilian floodplain of Paraíba do Sul around 25 years ago [53]. Sand extraction rates are substantially higher in Asia, with 59 million t extracted from the Lower Mekong River in Cambodia in 2020 [54], 42.9 million t per year in the Mekong Delta in Vietnam between 2018 and 2020 [55], and a staggering 236 million t in the Poyang Lake area in China in 2005–2006 [56]. Globally, the estimated annual extraction of 40 to 50 billion tons of sand corresponds to an average of 18 kg per person and day [57]. Our calculations indicate that the approximately 2.8 million residents of Bamako require, with only around 9 kg per person and day less than half of the global average [47].

A deepening of the Niger River resulting from sand mining around Bamako was also reported by Ferry et al. [29]. However, their findings indicated a slightly lower level of river depth reduction with average values of 0.18 to 0.82 m at three locations between 1982 and 2009/2010. This translates to an annual riverbed deepening of approximately 0.64 to 2.9 cm, compared to our observation of 2.9 to 4.0 cm. This difference may be attributed to two factors: Firstly, the recent acceleration of mining activities may have contributed to increased levels of river depth reduction. Secondly, our study focused on specific sand extraction hotspots, whereas Ferry et al. [29] examined longer stretches of the river. Comparatively, the river catchments of Vembanad Lake in India experienced with 7 to 15 cm per year (1980 to 2000) significantly stronger riverbed lowering [58]. Research conducted on the Poyang Lake in China revealed that sand mining resulted in the formation of sandpits with depths of 1.4 to 12 m compared with the surrounding bed surface [59].

Some interviewees have suggested that the construction of the Sélingué dam in 1982 and reduced rainfall levels in recent years are contributing factors to the decreasing river depth. However, upon closer examination, both explanations appear insufficient. Research conducted by Ferry et al. [29] revealed that sediment fill in the catchment area of the Sélingué dam of the River Niger remained relatively stable between 1964 and 2008, suggesting that it did not significantly impede the flow of sand. Instead, the dam facilitated navigability and, consequently, sand mining activities by augmenting the annual minimum flow of the Niger (increasing from an average of 42 m^3^/s between 1907 and 1981 to 96 m^3^/s from 1982 to 2010). Additionally, annual total rainfall in Bamako exhibited a slight increase between 1991 and 2020 [34]. Also, the frequency and intensity of extreme rainfall events grew between 1982 and 2019 [60]. Given that while sand input has remained relatively stable over the past decades, the increased riverbed incision may be primarily attributed to the increase in sand mining.

River damming and sand/gravel mining can lead to a reduction in downstream sediment delivery causing an erosion of channel beds, banks, and beaches [61]. In Vietnam, this phenomenon has adversely affected the stability of the Mekong Delta and its fisheries, as well as soil fertility along the Mekong River Basin [62]. However, it is important to note that due to the siltation occurring in the Sahel-Saharan triangle, which poses challenges for fishers and farmers [63], sand mining activities around Bamako did not result in a shortage of sedimentation downstream, neither in other Malian cities nor the neighboring countries or the Niger River Delta.

Our data indicate that sand mining activities did not affect turbidity of the Niger River. The measured turbidity range (10–92 NTU, average 26 NTU) confirm the results obtained by Sangare et al. [64] who reported around Bamako values of 2 to 91 NTU, with an average of 22 NTU. This contrasts with previous research on sand mining, which has indicated a major effect on water turbidity [65]. Balogun et al. [66] attributed an increase in water turbidity in the Lagos Harbor to sand mining activities. A study conducted on the Kelantan River, Malaysia, revealed a significant increase in turbidity levels attributed to sand mining activities, resulting in adverse conditions for aquatic life [12]. Similarly, Ashraf et al. [67] observed that turbidity levels peaked at sand dredging sites and gradually decreased with increasing distance downstream.

In a previous study around Bamako, Ferry et al. [29] observed an increasing undermining of riverbanks and an augmented water flow in Koulikoro for the past few decades, indicating morphological changes of the Niger River. The destruction of riverbeds and the adverse effects on aquatic fauna, as well as the deterioration of water quality and alterations in riverbed morphology, were also acknowledged by Dagno et al. [27] in their research conducted in Koursalé near Bamako. However, their findings indicated that 80% of the interviewees attributed the observed environmental effects to gold dredging, whereas only 20% associated them with sand mining. It further needs to be noted that respondents of our study may have provided overly positive assessments of sand mining activities, possibly influenced by personal involvement in the industry or connections to family or community members engaged in sand mining. The social desirability bias could have led to an understatement of mining effects on the environment and community.

Ferry et al. [29] also noted disruptions in fishing activities to sand mining. The reported proliferation of holes in the riverbed altered resting spots, degraded spawning grounds, and allowed fish to escape nets due to their inability to conform to the irregular shapes of the holes. Additionally, the constant movement of pirogue convoys hindered the installation of standing nets in the river channel. In Lagos State, Nigeria, Adesina and Adunola [68] reported that 56% of the fishermen perceived sand dredging as negatively affecting fishing activities, resulting in reduced fish catch, increased fish mortality, frequent escape of fish during fishing operations, prolonged fishing duration, and water contamination.

The sand mining industry triggered major changes in the local communities as it led to the formation of associations, the establishment of relationships with other livelihood strategies, and economic growth. This supports the argument of Franks [69], who stressed the potential for informal mining to contribute to the governance and sustainability of sand extraction while improving the livelihoods of the actors involved. Although it is not uncommon for sand miners to view their activities in an overly positive light [70] or euphemize bribes and licensing shortcuts [50], the examples provided by Franks [69] and our study are among the few instances where informal, artisanal sand mining systems are publicly recognized as beneficial, rather than illegal, destructive or criminal. Our findings, however, contradict Ganie and Bhat [21] who summarized for the BRICS countries, that local communities did not reap the benefits from sand mining, while bearing the negative externalities. Their study infers that exploitation of the common pool resource sand is prone to undergo the classical tragedy of the commons. This is supported by Katz-Lavigne et al. [71], who contended that the absence of durable institutions to regulate resource use, the lack of environmental and communal benefits challenge the extraction of sand as a common pool resource. Further challenges are the temporary and often unsustainable nature of the livelihood opportunities, and the predominant focus on commercial interests rather than the needs of user groups. In contrast to other common pool resources governed by Ostrom’s framework [72], this makes sand mining hard to regulate locally. Nevertheless, the typical negative outcomes of unlimited resource exploitation seem less prominent and unfold at a slower pace in the Niger River around Bamako than at other sand mining regions. Artisanal mining activities demonstrate adherence to several of Ostrom’s eight design principles, while other aspects do not align with her framework (Table 2).

**Table 2:** Ostrom’s design principles [71] applied to artisanal sand mining practices around Bamako, southwest Mali.

Design principles	Aligned components	Non-aligned components
1. Clearly defined boundaries	Newcomers must obtain approval from association heads to acquire a mining permission and designated sand collection site	Inclusive participation: Generally open to all; No constant mining point: extraction area increasing over time
2. Congruence between appropriation and provision rules and local conditions	Prohibition of mechanized mining in certain communities; Socioeconomic symbiosis with farming, fishing, and other livelihoods; Local solution approaches to address road degradation	No restrictions on extraction quantities and time; Extraction surpasses natural replenishment rates
3. Collective-choice arrangements	Collective determination of sand selling prices	Limited participation of women in decision-making processes
4. Monitoring	Association heads or their representatives are always present on-site	
5. Graduated sanctions	Monetary sanctions imposed for rule violations (e.g., underselling sand, denying pirogue passage of loaded tie-up, engaging in fights)	
6. Conflict-resolution mechanisms	Association heads or their representatives are always present on-site	
7. Minimal recognition of rights to organize	Very limited intervention by state organizations in sand mining operations	Associations often unrecognized by state bodies; Sand mining perceived as unregulated
8. Nested enterprises	Arrangements spanning multiple sites (e.g., one association overseeing three sand mining locations)	Sand mining associations and operations primarily locally organized, lacking regional or national centralization or arrangements

In recent years, mechanized mining systems, employing sand suction hoses and bulldozers, have been implemented at various locations at Koursalé, Makono, and Bancoumana, 60 km upstream of Bamako [27]. Their effects on sustainability parameters are multifaceted, displaying advantages and disadvantages: economically, mechanical sand mining is more productive, capable of yielding significant amounts of sand at a lower cost, potentially expediting infrastructure development. However, this economic advantage may not be well distributed, possibly exacerbating inequalities. On a social level, artisanal sand mining generates employment opportunities for thousands of laborers, including many unskilled workers, thereby alleviating poverty among families in the rural, peri-urban, and urban areas around Bamako. On the other hand, the working conditions associated with artisanal mining activities remain problematic, posing risks to the health and safety of those involved. From an environmental standpoint, manual sand mining presents a more environmentally friendly option compared to mechanical methods [46].

Despite the adverse environmental and social consequences associated with mechanized sand mining, its utilization is anticipated to grow in the coming years, potentially leading to a partial or complete replacement of traditional artisanal mining practices. In Dhaka, Bangladesh, the transition from manual to mechanized sand mining occurred in the mid-1990s due to the inability of the former to meet the rising demand, resulting in tension and collapsing labor opportunities, followed by a switch to port labor of sand [73]. Currently, Bamako still relies on an artisanal workforce for both sand extraction and unloading processes and any shift to mechanized mining would inevitably disrupt and transform the entire mining system. Coupled with public perceptions of siltation of the Niger River and the undervaluation of sand’s role in riverine protection, along with weak enforcement of governmental regulations and the burgeoning demand for sand driven by urbanization, an overexploitation of sand in the foreseeable future is likely.

The transition from artisanal to mechanized sand mining and its implications for sustainability parameters warrant further investigation. To take more informed decisions in the sector of sustainable sand mining, comparative research examining the environmental and social effects of these systems will provide valuable insights for policy makers and local stakeholders. This implies the need for a comprehensive study elucidating the extent and root causes of environmental alterations along the Niger River and considering the growing interplay of sand mining, gold dredging, damming, and fishing. Attention should also be paid to the increasing river depth, assessing both adverse and beneficial consequences and its interaction with erosion processes. Such research endeavors could facilitate more effective regulation of riverine activities, leveraging existing structures while imposing necessary regulations or limits on extraction to prevent the accelerated depletion in a tragedy of the commons scenario.

In addition, promoting the use of sustainable building materials as alternatives to sand could serve as a long-term measure to curb the demand for sand in and around Bamako, contributing to a more sustainable construction sector in the future.

## 5 Conclusions

Despite its predominantly artisanal and informal nature, sand mining around Bamako has witnessed a notable surge in recent years. Although its extraction rate far exceeds the natural replenishment rate, resulting in river incisions, the observed environmental effects seem relatively minimal compared with other areas of sand extraction. Despite its poor working conditions, sand mining provides a vital source of employment within local communities. It also has fostered an economic symbiosis among many farming and fishing families in the study areas. The anticipated shift towards mechanized sand mining raises concerns about growing environmental destruction and disruption of social dynamics within the existing mining framework. Future research should focus on assessing, supporting, and regulating artisanal sand mining activities in and around Bamako, taking the potential effects on the environment and the well-being of the communities into consideration.

## Supporting information

S1 File Overview of survey questions. (PDF)

S2 File Raw data of quantitative interviews. Column D: Usine Toch is in the Excel file listed as Baguineda.
(XLSX)

S3 File Truck count data and calculations. (XLSX)

S4 File Turbidity measurement data. (XLSX)

S5 File River depth measurements and retrospective data. (XLSX)
