# Supplementary material for: Social ecology of artisanal sand mining in the Niger River around Bamako, Mali
Source: PLoS One. 2025 Jan 30;20(1):e0318029. doi: 10.1371/journal.pone.0318029 (PMC11781661; doi:10.1371/journal.pone.0318029)
Supplement: S1 File — (PDF) [file pone.0318029.s001.pdf]

Questionnaire_Mali Farmer+Fisher+SandMiner

Introduction

1 Questionnaire ID & Accord sur la protection des données

____

2 Nom de la communauté

____

3 Lieu (GPS)

____

latitude (x.y °)

____

longitude (x.y °)

____

altitude (m)

____

accuracy (m)

____

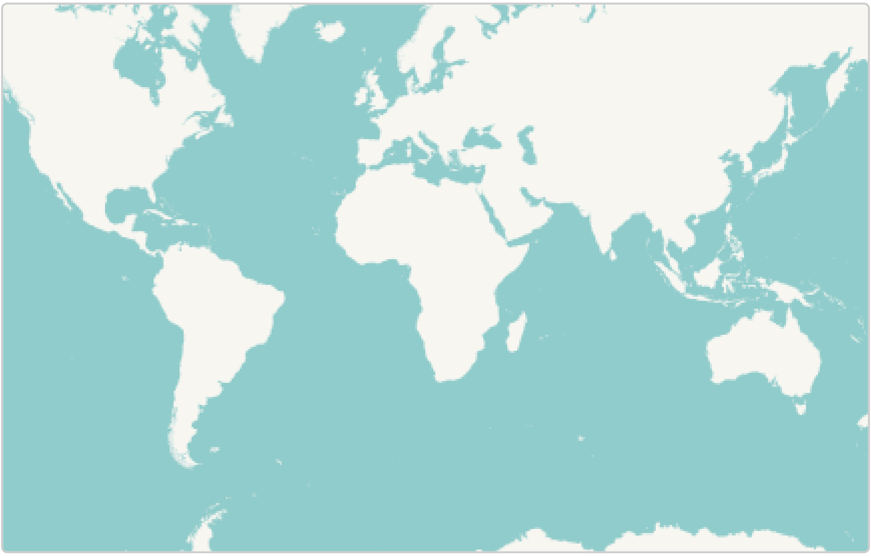

4 Lieu (GPS) manuel

____

5 Nom

____

6 Numéro de téléphone

____

7 Sexe

- ◯ Féminin
- ◯ Masculin
- ◯ Autre

8 Âge

____

9 Années de scolarité / plus haut niveau d'éducation

10 Taille du ménage (famille nuclaire)

11 Êtes-vous un agriculteur ?

- ◯ Oui
- ◯ Non
- ◯ Je l'étais

12 Pourquoi non plus?

Agriculture

13 Depuis combien d'années ?

14 Combien de champs cultivez-vous ?

» Culture

1

15 Quelle(s) culture(s) pratiquez-vous ?

16 Quelle est la taille de votre champ?

17 Arrosez-vous le champ ?

- ☐ Oui
- ☐ Non
- ☐ Précisez

18 Comment arrosez-vous votre champ?

19 Gardez-vous ou vendez-vous la récolte ?

- ☐ Garder
- ☐ Vendre
- ☐ Les deux
- ☐ Autre

20 Où vendez-vous la recolte?

____

21 À quel prix [unité et fréquence par an]?

____

22 Quels sont les rendements [unité et fréquence par an] ?

____

23 La taille totale de vos champs a-t-elle changé au cours des 10 dernières années ?

- ◯ Oui
- ◯ Non
- ◯ Je ne sais pas.

24 Comment et pourquoi ?

____

25 Qui est propriétaire des champs que vous cultivez ?

____

26 Quelle est la sécurité de vos droits d'accès ?

____

28 Quelles sont les difficultés de l'agriculture ?

____

29 Êtes-vous pêcheur ?

- ◯ Oui
- ◯ Non
- ◯ Je l'étais

30 Pourquoi non plus?

____

Fishing

31 Depuis combien d'années ?

____

32 Gardez-vous ou vendez-vous le poisson ?

- ☐ Garder
- ☐ Vendre
- ☐ Les deux
- ☐ Autre

33 Où vendez-vous?

34 Quelles espèces de poissons pêchez-vous ?

35 Quelle quantité pêchez-vous (saison sèche et saison des pluies) ?

38 Quels est le prix par kg ?

36 L'espèce et/ou la quantité ont-elles changé au fil du temps ?

- ◯ Oui
- ◯ Non
- ◯ Je ne sais pas

37 Pourquoi ?

39 Êtes-vous propriétaire ou locataire du bateau de pêche ?

- ◯ Propriétaire
- ◯ Locataire
- ◯ Autre

40 Combien payez-vous ?

41 Le louez-vous à d'autres pêcheurs ou à des mineurs de sable ?

- ◯ Oui
- ◯ Non
- ◯ Autre

42 À quel prix ?

43 Engagez-vous de la main-d'œuvre pour la pêche ?

- ◯ Oui
- ◯ Non
- ◯ Autre

44 Combien payez-vous ?

45 Quelles sont les difficultés de la pêche ?

46 Êtes-vous mineur de sable?

- ◯ Oui
- ◯ Non
- ◯ Je l'étais

47 Pourquoi non plus?

Mineur de sable

» Introduction

48 Depuis combien d'années ?

---

49 Avez-vous eu un autre emploi avant ? Si oui, lequel ?

---

50 Pourquoi avez-vous décidé de travailler dans les mines de sable ?

---

51 Quelle est votre tâche exacte ? (Patron, plonger, décharger la pirogue, charger le ben, conduire)

- ☐ Patron
- ☐ Plonger
- ☐ Operateur de pirogue
- ☐ Déchargeur/euse de la piroque
- ☐ Chargeur du ben
- ☐ Conduire le ben
- ☐ Chargeur des charrettes
- ☐ Tamiser le gravier
- ☐ Collecter le sable avec seau
- ☐ Collecter le sable autour des pirogues
- ☐ Autre / Explication

52 Autre

---

53 Quels sont les aspects négatifs de votre travail ?

---

54 Quels sont les aspects positifs de votre travail ?

---

55 Considérez-vous votre travail comme dangereux ? Expliquez.

» Ampleur de l'extraction

56 Quand commencez-vous et terminez-vous votre travail ?

57 Combien de jours par semaine travaillez-vous ?

Quel est le type d'extraction que vous faites?

- ☐ Pirogue
- ☐ Charette
- ☐ Benne (directement)
- ☐ Seau
- ☐ Autre / Explication

Autre / Explication

58 Nombre de personnes avec qui vous travaillez en total

59 Lieu de l'extraction

60 Comment a le lieu changé au fil du temps?

Est-ce que chacun peut choisir le lieu de l'extraction lui-même librement?

61 Profondeur de l'extraction (m)

62 Ca dûre combien de temps pour aller-retour (aller, charger, retourner, décharger)?

Ca dûre combien de temps pour charger une charette?

Ca dûre combien de temps pour charger un ben?

63 Quel type de sable exploitez-vous?

- [ ] Petit
- [ ] Moyen
- [ ] Grand
- [ ] Autre / Explication

64 Expliquez "Autre"

65 Prenez-vous aussi du gravier?

- ( ) Oui
- ( ) Non
- ( ) Explication

66 Expliquez

67 Est-ce qu'il y a une liason avec l'extraction de l'or?

- [ ] Oui, je cherche aussi de l'or
- [ ] Oui, je prends le sable/gravier de l'extraction de l'or
- [ ] Non
- [ ] Autre / Explication

68 Expliquez

69 Combien de pirogues faut-il pour charger un ben?

Combien des charrettes faut-il pour charger un ben?

70 Quelle quantité de sable exploitez-vous pendant la saison sèche (par jour/semaine) [pirogue / ben / charrette] ?

71 Quelle quantité de sable exploitez-vous pendant la saison des pluies (par jour/semaine) [pirogue / ben / charrette]?

Quelle quantité de gravier exploitez-vous pendant la saison sèche (par jour/semaine) [pirogue / ben / charrette] ?

Quelle quantité de gravier exploitez-vous pendant la saison des pluies (par jour/semaine) [pirogue / ben / charrette] ?

72 Comment c'est évolué avec le temps (aujourd'hui, il y a 5 ans, il y a 10 ans) ?

73 Combien de mineurs de sable travaillent ici ?

74 Comment c'est évolué avec le temps ?

75 A votre avis, combien de ben par jour sont exploités sur ce site (pendant la saison sèche et la saison de pluies) ?

Pensez-vous que la quantité de l'extraction surpasse le remplissage naturel dans le fleuve?

- [ ] Oui
- [ ] Non
- [ ] Je ne sais pas
- [ ] Autre / Explication

Autre / Explication

» Structure des coûts et des revenus

76 Qui achète le sable ?

77 Quelle est la destination du sable (région / quartier) ?

78 Est-ce qu'il faut payer quelque chose à l'origin du sable pour la communauté?

- ◯ Oui
- ◯ Non

79 Combien? (prix par pirogue / charrette/ ben ou prix par temps) ?

80 Quel est le prix de vente du sable (par tailleur) ?

81 Comment c'est évolué avec le temps (aujourd'hui, il y a 5 ans, il y a 10 ans) ?

Quel est le prix de vente du gravier (par tailleur) ?

82 Quels sont les coûts initiaux pour commencer l'exploitation du sable ?

83 Quels sont vos coûts récurrents ?

84 Combien gagnez-vous à la fin de la journée (profit / salaire (par unité et total)) ?

» Réglementation

85 Faites-vous partie d'une association ?

- ◯ Oui
- ◯ Non
- ◯ Explication

86 Expliquez

87 Comment affecte ça vos activités ?

88 Quelles règles régissent les activités d'extraction de sable dans cette communauté (institution ou règles communales) ?

89 Avez-vous besoin d'un permis d'extraction de sable ? Expliquez

90 Est-ce qu'il y a une extraction de sable illégale ? Expliquez

Autre boulot

91 Avez-vous un autre boulot?

- ◯ Oui
- ◯ Non

Lequel?

Est-ce qu'il y a une influence de l'extraction de sable à votre boulot?

Effets sur l'environnement

92 À cause de l'extraction du sable, a la rivière changé

- ☐ La qualité de l'eau
- ☐ La quantité (le niveau) de l'eau
- ☐ La force de l'eau
- ☐ Explication / Autre
- ☐ Aucune de ces réponses

Autre

95 L'extraction de sable a-t-elle provoqué l'érosion des terres dans la rivière ?

96 L'extraction de sable a-t-elle une influence sur les arbres / les plantes / les animaux sauvages terrestres / les oiseaux ? Si oui: Lequel et comment?

Effets sur la communauté

98 L'extraction de sable cause-t-elle des problèmes de la pollution?

- [ ] Bruit
- [ ] Poussière
- [ ] Eau potable
- [ ] Explication / Autre
- [ ] Aucune de ces réponses

99 Explication / Autre

100 L'extraction de sable a-t-elle une influence sur l'infrastructure de la communauté (pirogues, routes, ponts) ? Expliquez

101 L'extraction de sable a-t-elle une influence sur la santé ou du bien-être des membres de la communauté ? Expliquez

Effets sur l'agriculture

103 Pensez-vous que l'extraction de sable influence les activités agricoles ?

- ○ Oui
- ○ Non
- ○ Je ne sais pas.

104 Expliquez

105 L'extraction du sable influence-t-elle

- [ ] La taille / disponibilité des terres agricoles
- [ ] Le rendement (quantité de produits) ?
- [ ] La qualité des produits
- [ ] La disponibilité de la main-d'œuvre
- [ ] Explication / Autre
- [ ] Aucune de ces réponses

Autre

Effets sur la pêche

109 Pensez-vous que l'extraction de sable influence les activités de pêche ?

- ○ Oui
- ○ Non
- ○ Je ne sais pas

110 Expliquez

---

111 L'extraction de sable influence-t-elle

- [ ] La quantité des poissons
- [ ] Le type des poissons
- [ ] Les autres espèces
- [ ] La disponibilité des pirogues
- [ ] La disponibilité de la main-d'œuvre
- [ ] Explication / Autre
- [ ] Aucune de ces réponses

Explication / Autre

Perspectives

116 Pensez-vous que les activités d'extraction de sable vont se poursuivre dans votre communauté ?

---

117 Voulez-vous que vos enfants deviennent agriculteurs/pêcheurs/mineur de sable ?

- [ ] Agriculteur
- [ ] Pêcheur
- [ ] Mineur de sable
- [ ] Non
- [ ] Comme ils veulent / Autre

118 Comment la politique pourrait-elle soutenir les agriculteurs/pêcheurs/mineurs de sable ?

---

121 Dernière question: Est-ce qu'il y a une autre chose à ajouter?
